# EP2 and EP4 in Alzheimer’s Disease: Cell-Dependent Signalling and Implications for Selective Targeting

**DOI:** 10.3390/ijms27209190

**Published:** 2026-10-16

**Authors:** Xi Zhao, Youjia Zeng, Zhenyan Song, Jiawei He, Qijun He, Han Lin, Lu Liu, Wenjing Yu, Shaowu Cheng

**Affiliations:** 1 School of Integrated Chinese and Western Medicine, Hunan University of Chinese Medicine, No. 300 Xueshi Road, Yuelu District, Changsha 410208, China; 20242120@stu.hnucm.edu.cn (X.Z.);; 2 Key Laboratory of Hunan Province for Integrated Traditional Chinese and Western Medicine on Prevention and Treatment of Cardio-Cerebral Diseases, Hunan University of Chinese Medicine, Changsha 410208, China; 3 Medical School, Hunan University of Chinese Medicine, No. 300 Xueshi Road, Yuelu District, Changsha 410208, China

**Keywords:** Alzheimer’s disease, prostaglandin E2, EP2, EP4, microglia, ageing, myeloid metabolism

## Abstract

Prostaglandin E2 (PGE_2_) signalling influences amyloid-β (Aβ) processing, myeloid function, and neuronal injury in Alzheimer’s disease (AD). Although EP2 and EP4 share Gs–cyclic adenosine monophosphate (cAMP) signalling, their cellular effects differ. This review integrates genetic, pharmacological, ageing, and AD-model evidence to assess receptor-selective targeting. EP2 deletion enhances myeloid Aβ handling and reduces microglia-mediated neuronal injury in experimental systems, providing a rationale for selective EP2 inhibition. In aged mice, PGE_2_–EP2 signalling redirects glucose towards glycogen storage and restricts myeloid mitochondrial respiration; benefits from a predominantly peripheral antagonist suggest that some age-related outcomes may improve without high brain exposure. Chronic antagonist studies in AD models, however, show variable effects on inflammation, amyloid burden, and behaviour. EP4 exhibits contrasting cell-dependent actions: activation supports microglial Aβ uptake and inflammatory control, whereas signalling in some amyloid precursor protein (APP)-expressing cells promotes APP internalisation and Aβ production. Human AD evidence remains mainly tissue-based; EP2 antagonist testing has involved healthy volunteers, not patients with AD. These findings indicate that receptor selectivity alone is insufficient to predict therapeutic outcomes. Further studies should combine cell-specific receptor manipulation, measurements of central and peripheral exposure, and separate assessments of Aβ production, clearance, tissue injury, and functional outcomes.

## 1. Introduction

Alzheimer’s disease (AD) is the most common cause of dementia, accounting for approximately 60–70% of cases worldwide [1]. Progressive loss of cognition and daily functioning increases dependence on care and places psychological, physical, and financial burdens on family caregivers [2]. Dementia-related medical care, long-term care, and informal caregiving also impose substantial costs on health and social care systems [3]. The development and progression of AD involve multiple interacting pathological processes, including amyloid-β (Aβ) deposition, tau abnormalities, synaptic injury, and persistent neuroimmune alterations [1,4]. Microglia recognize and clear Aβ, but sustained activation can release mediators that damage synapses and neurons [4]. In animal models of AD, modulation of microglial prostaglandin E2 (PGE_2_) receptor signalling affects Aβ handling, inflammatory responses, and synaptic and memory outcomes [5,6]. These findings place microglial prostaglandin signalling at the intersection of Aβ clearance and inflammatory injury.

Recent anti-amyloid trials provide a clinical context for mechanism-directed treatment. In Clarity AD, lecanemab slowed decline on cognitive and functional measures over 18 months and substantially reduced amyloid burden, with infusion reactions and amyloid-related imaging abnormalities [7].

Donanemab likewise slowed clinical progression in early symptomatic AD with amyloid and tau pathology, while producing amyloid-related imaging abnormalities and infusion reactions [8].

Early studies of prostaglandin-related inflammation in AD targeted cyclooxygenase (COX). One year of rofecoxib or naproxen treatment did not slow cognitive decline in patients with mild-to-moderate AD [9]. Cognitive analyses from the Alzheimer’s Disease Anti-inflammatory Prevention Trial (ADAPT) likewise found no benefit from naproxen or celecoxib, with worse performance on some measures [10]. COX inhibition affects the synthesis of several prostaglandins whose receptors have distinct, sometimes opposing, functions [11]. Receptor-selective interventions offer a way to separate these downstream actions.

PGE_2_ acts through four E-prostanoid (EP) receptor subtypes, EP1, EP2, EP3, and EP4 [11]. EP2 and EP4 both stimulate G_s_-dependent cyclic adenosine monophosphate (cAMP) production. Nevertheless, EP2 loss increases microglial Aβ uptake and clearance and reduces Aβ-induced paracrine neurotoxicity [5,12]. Microglial EP4 activation also promotes Aβ uptake, while suppressing Aβ-associated inflammatory responses [6]. Their shared ligand and signalling pathway therefore conceal important differences in cellular function.

Ageing adds a metabolic dimension to these receptor effects. In aged mice, increased PGE_2_–EP2 signalling alters glucose utilisation in myeloid cells, whereas EP2 inhibition restores glucose flux and mitochondrial respiration and improves synaptic and memory outcomes. Comparable benefits obtained with a predominantly peripheral antagonist further indicate that therapeutically relevant EP2 signalling is not confined to CNS-resident myeloid cells [13]. Recent reviews have largely discussed prostaglandins as mediators and therapeutic targets in the inflamed CNS or examined EP2 as an individual pharmacological target [11,14,15]. Rather than considering receptor subtype alone, the present review evaluates EP2 and EP4 within a common framework defined by cellular source, Aβ production versus uptake and clearance, and central versus peripheral drug action. This framework helps explain why systemic and cell-restricted receptor manipulations can produce different outcomes and clarifies the evidence required to evaluate receptor-selective interventions in AD.

Literature search. PubMed, Europe PMC, and ClinicalTrials.gov were searched through 1 October 2026. Search terms included combinations of “PGE_2_,” “PTGER2” or “EP2,” “PTGER4” or “EP4,” “Alzheimer’s disease,” “amyloid-β,” “microglia,” “ageing,” “agonist,” “antagonist,” “pharmacokinetics,” and “clinical trial.” The reference lists of relevant articles were also screened. Original studies and reviews were included when they addressed EP2/EP4 biology, AD-related mechanisms, ageing, or receptor pharmacology. Studies conducted outside AD models were retained when they provided relevant mechanistic or pharmacological evidence.

## 2. PGE_2_ Homeostasis and Its Alterations in AD

Local PGE_2_ availability is jointly regulated by synthesis, transmembrane transport, and metabolic inactivation [16,17,18]. AD-associated changes in synthetic enzymes and transport proteins, together with differences between brain-tissue and cerebrospinal-fluid (CSF) PGE_2_ measurements, frame the ligand supply available to EP receptors [19,20,21,22,23].

### 2.1. PGE_2_ Synthesis, Transport, and Inactivation

Arachidonic acid released from membrane phospholipids is converted by COX-1 and COX-2 to prostaglandin H2 (PGH_2_), the common precursor of prostaglandins, and is subsequently converted to PGE_2_ by prostaglandin E synthases. Microsomal prostaglandin E synthase-1 (mPGES-1) is an important terminal synthase under inflammatory conditions. In contrast to COX inhibition, which acts upstream of the synthesis of multiple prostaglandins, targeting mPGES-1 restricts the intervention more specifically to PGE_2_ production [18].

Studies of human brain tissue have shown that mPGES-1 protein levels are increased in the middle frontal gyrus of patients with AD compared with age-matched controls, with expression detected in neurons, microglia, astrocytes, and endothelial cells [19]. A separate study reported increased plaque-associated mPGES-1 expression in AD cortex and in Tg2576 mice. Genetic deletion of mPGES-1 reduced microglial accumulation around plaques and improved learning performance in the mouse model [24]. PGE_2_ production around plaques thus involves several cell populations.

In 5×FAD mice, Wang et al. found that COX-1 increased with age, was predominantly localised to microglia, and accompanied elevated brain PGE_2_. COX-1 deletion reduced neuroinflammation and cognitive impairment. An EP2 agonist reversed some microglial changes in the COX-1-deficient background. The EP2 antagonist TG4-155 inhibited PGE_2_-induced inflammasome signalling in BV2 cells; the in vivo cognitive results were obtained with COX-1 deletion [25].

Extracellular PGE_2_ is taken up by the prostaglandin transporter PGT (SLCO2A1/OATP2A1) and oxidised intracellularly by 15-hydroxyprostaglandin dehydrogenase (15-PGDH). Uptake and inactivation limit extracellular ligand availability and signalling duration [16,17,26].

PGE_2_ transport includes export as well as reuptake. Wen et al. resolved human ATP-binding cassette subfamily C member 4 (ABCC4) bound to PGE_2_ and identified binding-site interactions involved in PGE_2_-stimulated ATPase activity. These structures describe substrate recognition on the export side, complementing the SLCO2A1 uptake pathway [27].

In the middle frontal gyrus of human AD brain, PGT is detectable in neurons, microglia, and astrocytes, whereas its protein abundance in brain homogenates is lower than that in age-matched controls [20]. The reduced transporter abundance and increased mPGES-1 reported in separate cohorts suggest changes in ligand supply and removal. Matched measurements of these proteins and extracellular PGE_2_ would establish their combined effect more directly.

### 2.2. PGE_2_ Availability and Receptor-Mediated Responses in AD

PGE_2_ measurements differ across AD cohorts and sampling compartments. Early CSF studies reported elevated PGE_2_, while longitudinal observations related its levels to cognitive impairment and survival [21,28]. In more recent lipidomic studies, Do et al. found lower CSF PGE_2_ levels in patients with mild cognitive impairment (MCI) and AD than in individuals with subjective cognitive impairment, although after age matching the difference remained only for the MCI group. Ahmad et al. reported an association between PGE_2_ and CSF Aβ42, but this association did not survive correction for multiple comparisons [23,29]. In contrast, lipidomic analysis of postmortem dorsolateral prefrontal cortex showed increased PGE_2_ in AD dementia, with higher levels associated with poorer cognitive performance and greater histopathological AD burden; some of these clinical and pathological associations were further modified by apolipoprotein E ε4 (APOE4) status [22]. These studies differ in diagnostic groups, age matching, sampling compartment, and analytical methods.

CSF and brain homogenates sample different PGE_2_ pools. Neither resolves extracellular concentrations around individual responding cells, making compartment and sampling conditions essential to interpretation.

Ligand abundance alone also leaves the responding receptor unresolved. EP2 and EP4 share PGE_2_ but differ in coupling, trafficking, and cellular effects. These receptor properties connect altered PGE_2_ availability to microglial responses. PGE_2_ homeostasis and the principal signalling features of EP2 and EP4 are summarised in Figure 1.

## 3. EP2 and EP4 Signalling and Microglial Responses

EP2 and EP4 are encoded by *PTGER2* and *PTGER4*, respectively. Both receptors can couple to G_s_ proteins, activate adenylyl cyclase, and increase intracellular cAMP [30,31]. Their effects depend on receptor coupling and trafficking as well as the downstream effectors available in the responding microglia.

### 3.1. G-Protein Coupling and Receptor Trafficking of EP2 and EP4

In HEK293 cells expressing EP2 or EP4, Fukushima et al. found greater PGE_2_-induced cAMP accumulation through EP2 when phosphodiesterase activity was not inhibited. Pharmacological modelling supported preferential G_s_-related signalling through EP2 and a stronger G_i_-related component through EP4 in this expression system [30]. The comparison also shows why phosphodiesterase inhibition and assay timing matter when measuring receptor-dependent cAMP.

Huang et al. resolved EP4–G_s_ and EP4–G_i_ complexes bound to PGE_2_ or synthetic agonists. Different ligand-binding conformations propagated through the transmembrane region to favour distinct G-protein interactions [31]. These structures establish EP4 coupling capacity; the relative G_s_ and G_i_ contributions in microglia require cell-specific functional measurements.

The EP2 side of this structural comparison is supported by agonist-bound EP2–G_s_ complexes. Qu et al. resolved complexes with PGE_2_, taprenepag, and evatanepag and identified an unconventional activation mechanism and a role for helix 8 in G_s_ coupling [32].

EP4 has a relatively long cytoplasmic C-terminal tail. In heterologous-expression experiments, it rapidly internalised after agonist stimulation, whereas EP2 showed limited agonist-induced internalisation under comparable conditions. Truncating the EP4 C terminus markedly reduced internalisation [33]. EP4 internalisation involves β-arrestin, dynamin, and clathrin-coated vesicles. PGE_2_ also rapidly activates mitogen-activated protein kinase (MAPK), but inhibition of receptor internalisation does not concomitantly block MAPK activation, indicating that receptor trafficking and at least some downstream signalling events can be regulated separately [34]. In cultured dorsal root ganglion neurons, internalised EP4 preferentially recycled to the plasma membrane after agonist stimulation, contributing to enhanced receptor sensitivity [35].

Walker et al. identified constitutive EP2 internalisation through a dynamin-dependent, β-arrestin-independent pathway, with trafficking to early and very early endosomes. Inhibiting this process altered agonist-induced cAMP and Ca^2+^ signals in an agonist-dependent manner [36]. The experiments, conducted mainly in heterologous-expression systems and human myometrial cells, distinguish constitutive trafficking from the limited agonist-induced internalisation described previously.

Together, these studies show that receptor internalisation and downstream signalling are not interchangeable measures. Constitutive EP2 trafficking modulates agonist-dependent cAMP and Ca^2+^ responses, whereas EP4 internalisation can be experimentally dissociated from MAPK activation. In microglia, the functional output also depends on the cAMP effectors engaged and on stimulation-induced changes in receptor expression.

### 3.2. cAMP Effectors and Microglial State Dependence

In microglia, cAMP can regulate cellular responses through effectors including protein kinase A (PKA) and exchange proteins directly activated by cAMP (Epac). In classically activated primary rat microglia, Quan et al. found that EP2 stimulation increased expression of COX-2, inducible nitric oxide synthase (iNOS), interleukin (IL)-6, and IL-1β, while reducing tumor necrosis factor (TNF)-α, IL-10, and C–C motif chemokine ligands 3 and 4 (CCL3 and CCL4). PKA inhibition had little effect on most of these changes, whereas Epac activation reproduced several of the effects of EP2 stimulation [37]. EP2 stimulation therefore changed individual inflammatory mediators in different directions within the same experiment.

PKA also contributes to some EP2-associated inflammatory responses. In BV2 cells, Wang et al. reported that PGE_2_ increased cAMP and activated PKA and nuclear factor κB (NF-κB) signalling together with NLR family pyrin domain containing 3 (NLRP3) inflammasome-related proteins; the EP2 antagonist TG4-155, the PKA inhibitor H89, and an NF-κB inhibitor each attenuated the corresponding responses [25]. The BV2 and primary-microglial experiments used different stimuli and endpoints. They identify PKA- and Epac-associated responses, respectively, rather than a fixed choice of effector across microglial states.

The duration of stimulation can also alter the receptor context in microglia. Bonfill-Teixidor et al. found that microglia not exposed to sustained lipopolysaccharide (LPS) stimulation predominantly expressed EP4, with lower levels of EP2, and that EP4 accounted for most of the PGE_2_-mediated suppression of early LPS-induced TNF-α production. Following LPS stimulation, EP2 and EP4 expression changed in opposite directions: EP2 mRNA increased whereas EP4 mRNA decreased, followed by a further reduction in EP4 immunoreactivity, increased EP2 protein expression, and greater perinuclear localisation of EP2, together with changes in the regulation of inflammatory and metabolic genes [38]. Sustained stimulation thus changes the receptor repertoire on which subsequent PGE_2_ signals act. This time-dependent shift was demonstrated under LPS stimulation.

Hansen et al. studied PGE_2_ responses in the context of multiple sclerosis. Lesion microglia showed increased EP2 expression alongside reduced tissue EP4. In human induced pluripotent stem cell (iPSC)-derived microglia, resting and cells activated with LPS and interferon-γ (IFN-γ) responded differently to PGE_2_ and receptor blockade. EP2 blockade attenuated part of the inflammatory transcriptional response in resting cells; in activated cells, receptor-blockade experiments implicated EP2 in some expression features associated with inflammatory resolution [39]. These transcriptional observations extend state dependence to human-derived microglia. Phagocytosis, survival, and neuronal-injury endpoints were not established by the expression profiles.

The receptor and effector studies explain why cAMP elevation has limited value as a stand-alone predictor of microglial function. For AD, the decisive tests concern Aβ processing and clearance, neuronal injury, and myeloid metabolism.

## 4. Effects of EP2 on Aβ Homeostasis, Microglial Function, and Tissue Injury

EP2 participates in both Aβ production and microglial clearance and also influences microglia-mediated neuronal injury [5,12,40,41,42,43].

### 4.1. APP Processing and Aβ Production

PGE_2_ can influence amyloid precursor protein (APP) expression and processing, with the contribution of EP2 varying according to cellular context [40,41]. In cultured rat microglia, Pooler et al. found that PGE_2_ increased full-length APP protein together with intracellular cAMP. The EP2 agonist butaprost produced a similar effect, whereas PKA inhibition attenuated the PGE_2_-induced increase in APP [41]. Hoshino et al. further showed that PGE_2_ increased Aβ release from HEK293 and SH-SY5Y cells expressing mutant APP, although the receptor contribution differed between the two cell types: the response in HEK293 cells was predominantly EP4-dependent, whereas both EP2 and EP4 were involved in SH-SY5Y cells. A cAMP analogue reproduced this effect, and secretase assays showed increased γ-secretase activity [40]. Pooler et al. [41] measured APP abundance; Hoshino et al. [40] directly examined Aβ release and proteolytic processing.

Changes in brain Aβ in vivo are also influenced by age and oxidative injury. Liang et al. found that global EP2 deletion reduced Aβ40, Aβ42, and plaque burden in APPswe/PS1ΔE9 mice with established amyloid pathology and was accompanied by lower lipid peroxidation and reduced APP β-C-terminal fragments. The same changes in Aβ were not observed in younger mice before plaque formation. Direct treatment of primary cortical neurons or APP-expressing Chinese hamster ovary (CHO) cells with an EP2 agonist likewise failed to increase Aβ release [42]. The accompanying oxidative injury and β-C-terminal-fragment changes support an indirect contribution through APP processing. Global EP2 loss also enhances myeloid Aβ handling, so the tissue Aβ result incorporates effects on both production-related pathways and clearance [5,12,42].

### 4.2. Microglial Uptake and Clearance of Aβ

Primary-cell and ex vivo studies identify EP2 as a restraint on microglial Aβ handling. Shie et al. found greater Aβ phagocytosis and clearance of deposits from human AD brain sections by EP2-deficient mouse microglia, together with reduced paracrine neurotoxicity [12]. Consistent with these deletion experiments, PGE_2_ and butaprost reduced fluorescent Aβ uptake in cultured rat microglia [44]. Subsequent work linked this effect to the scavenger receptor CD36 (cluster of differentiation 36). EP2 inhibition increased microglial CD36 expression, whereas EP2 activation reduced it. Stimulation of Toll-like receptor (TLR) 3, TLR4, or TLR7 decreased cell-surface CD36 and Aβ42 phagocytosis, and an EP2 antagonist partially reversed these changes; restoration of phagocytosis was largely dependent on CD36 [43].

Johansson et al. used different genetic strategies for distinct endpoints. Global EP2 deletion increased microglial recruitment around early plaques in APP-PS1 mice. Cd11b-Cre-mediated myeloid deletion improved clearance of acutely injected Aβ and attenuated synaptic and memory deficits; complementary experiments identified increased insulin-like growth factor 1 (IGF1) and related protective signalling [5]. In a bone marrow transplantation study, APPswe/PS1ΔE9 mice receiving EP2-deficient bone marrow had a lower brain Aβ burden than mice receiving wild-type marrow [45]. The selective EP2 antagonist compound 52 also enhanced macrophage-mediated clearance of amyloid plaques from ex vivo brain sections in a dose-dependent manner [46]. The transplantation experiment included myeloablation and brain entry of marrow-derived cells, while compound 52 was tested with macrophages in an ex vivo clearance assay [45,46].

Long-term pharmacology gives a less uniform plaque outcome. In the 2021 two-hit LPS-plus-5×FAD study, TG11-77.HCl reduced inflammatory responses in females despite a modest increase in Congo red-positive plaque area [47]. In the 2026 study on a B6SJL background, glial activation declined in both sexes, but lower inflammatory transcription and plaque burden were observed mainly in males [48]. Thus, lower glial reactivity was shared across treatment-responsive cohorts, whereas plaque responses varied [47,48].

Uptake assays measure a cellular function; chronic antagonist studies measure accumulated tissue pathology and behaviour [12,43,44,47,48]. The 2026 study found target-quadrant preference in the water-maze probe trial, most clearly in males, but no significant between-group learning difference during training or improvement in Y-maze alternation [48]. Section 7 examines the treatment schedules and behavioural results in detail.

### 4.3. Microglial Metabolism, Oxidative Neurotoxicity, and Cell Fate

Metabolism provides another route through which EP2 can alter myeloid function. AD-related experiments link EP2 deletion to improved Aβ handling and IGF1-related protective signalling [5]. In aged mice, Minhas et al. showed that EP2 inhibition reversed glucose diversion into glycogen and restored mitochondrial respiration [13]. Section 6 addresses this metabolic mechanism and its peripheral contribution.

Microglia–neuron co-culture studies provide direct evidence that EP2 contributes to neuronal injury. Shie et al. showed that EP2 deletion blocked neurotoxicity induced by LPS-activated microglia and reduced induction of iNOS and COX-2. Deletion of microglial iNOS, pharmacological inhibition of COX-2, or addition of superoxide dismutase and catalase likewise abolished neurotoxicity [49]. Experiments using Aβ stimulation similarly showed that EP2-deficient microglia could exhibit enhanced phagocytosis while causing less paracrine neuronal injury [12]. Cytokine levels did not fall uniformly [49]. Yousif et al. instead examined inflammatory-mediator release and MAPK phosphorylation, which butaprost reduced after poly(I:C) or LPS stimulation in primary microglia and hippocampal slices [50]. The distinction is between direct neuronal-injury assays and inflammatory-signalling endpoints [49,50].

Reactive oxygen species are also involved in the effects of EP2 on microglial survival itself. Co-culture experiments showed that scavenging reactive oxygen species attenuated EP2-associated neurotoxicity [49]. Fu et al. further found in cultured rat microglia that combined LPS and IL-13 stimulation initially induced microglial activation and was followed by EP2-dependent delayed cell death; treatment with an EP2 agonist alone could also induce cell death. Both an EP2 antagonist and reactive oxygen species scavengers attenuated this process. The study also identified caspase-1-associated effects and caspase-3 activation, whereas induction of heme oxygenase-1 or provision of its product carbon monoxide was protective [51]. These culture experiments establish a role in activated microglial survival; the consequences for chronic Aβ clearance remain unresolved.

The most consistent EP2 findings concern enhanced myeloid Aβ handling and reduced paracrine neuronal injury after receptor loss [5,12,43,49]. Plaque and behavioural responses to chronic antagonism are more dependent on model and treatment context [47,48].

## 5. Cell-Dependent Effects of EP4 on Microglial Responses and Aβ Homeostasis

EP4 regulates inflammatory responses and Aβ uptake in microglia [6,52], whereas in some APP-expressing cells it promotes amyloidogenic APP processing and Aβ production [40,53]. These effects make cellular source central to interpreting EP4 function. Figure 2 contrasts these cell-dependent EP4 effects with the myeloid effects of EP2 inhibition.

### 5.1. Microglial EP4 Limits Inflammation and Promotes Aβ Handling

The inhibitory effect of EP4 on microglial inflammatory responses has been observed under both LPS and Aβ stimulation. Shi et al. showed that selective EP4 activation reduced LPS-induced inflammatory gene expression in the hippocampus and in isolated adult microglia. In cultured microglia, this effect was accompanied by reduced phosphorylation of protein kinase B (Akt) and IκB kinase (IKK) and decreased nuclear translocation of NF-κB subunits [52]. Woodling et al. further reported that EP4 activation reduced Aβ42-induced inflammatory mediators and increased uptake of labelled Aβ42 by BV2 cells. Transcriptomic analysis of primary microglia showed that EP4 activation opposed multiple Aβ-induced expression changes involving genes related to interferon regulatory factors 1 and 7 (IRF1 and IRF7) and NF-κB [6].

Conditional deletion experiments in vivo yielded corresponding results. Following systemic LPS challenge, myeloid EP4 deletion increased brain lipid peroxidation and inflammatory gene expression [52]. In APPswe/PS1ΔE9 mice, EP4 deletion using a CD11b-Cre strategy likewise increased early inflammatory responses, oxidative protein modifications, and Aβ deposition, whereas differences in inflammation and Aβ were less pronounced at the later stage [6]. This strategy affects both microglia and peripheral myeloid cells. In addition, the early- and late-stage cohorts consisted of 5-month-old female mice and 9-month-old male mice, respectively, so the apparent stage-related differences are also confounded by sex [6].

Human studies provide additional information on EP4 expression. Woodling et al. found lower cortical EP4 protein levels in both MCI and AD than in age-matched controls, although protein quantification included only three individuals per group. Immunostaining detected EP4 in neurons and cells with glial morphology, with the overall reduction being most apparent in neurons [6]. Transcriptomic profiling of human microglia by Hsiao et al. detected *PTGER4* and showed higher expression in subcortical white-matter microglia than in cortical gray-matter microglia; this regional comparison was not an analysis of AD stage [54].

### 5.2. EP4 Can Promote Aβ Production in APP-Expressing Cells

In mutant APP-expressing cells, Hoshino et al. linked EP4 activation to increased cAMP, γ-secretase activity, and Aβ release [40]. Subsequent work connected EP4 to APP internalisation. Global EP4 deletion in APP23 mice reduced plaques and neuronal and synaptic injury. Oral AE3-208 lowered brain Aβ, inhibited APP internalisation and γ-secretase activation, and improved the cognitive measures assessed [53].

The cell-based production experiments and myeloid conditional-deletion experiments implicate different components of Aβ homeostasis [6,40]. APP-expressing cells provide a production mechanism, while myeloid EP4 supports inflammatory control and Aβ uptake; global EP4 inhibition incorporates both cellular domains [6,40,53]. Differences in model and age further contribute to the divergent animal outcomes.

### 5.3. EPRAP-Dependent Regulation of Inflammation

EP4 receptor-associated protein (EPRAP) shows different inflammatory functions in macrophages and microglia. In bone marrow-derived macrophages and RAW264.7 cells, Minami et al. found that EPRAP interacts with NF-κB1 p105/p50. Through EPRAP, PGE_2_–EP4 signalling attenuated LPS-induced phosphorylation and degradation of p105 and limited subsequent MAPK kinase 1/2 (MEK1/2) activation and the production of selected inflammatory mediators [55]. In contrast, Fujikawa et al. found that EPRAP deficiency reduced LPS-induced TNF-α and CCL2 expression in cultured microglia and attenuated phosphorylation of MAPK kinase 4 (MKK4), c-Jun N-terminal kinase (JNK), and p38. In an excitotoxicity model, EPRAP deficiency also reduced microglial activation and neuronal death [56].

A subsequent study in J20 mice extended these observations to AD-related pathology. EPRAP deficiency reduced microglial accumulation and expression of *Cd68* and TNF-α in the prefrontal cortex and hippocampus, consistent with the attenuated inflammatory response observed previously [56,57]. Cortical and hippocampal Aβ40 and Aβ42, spatial memory, and hyperactivity were unchanged. EPRAP deletion corrected abnormally reduced anxiety-like behaviour [57]. These results separate inflammatory and anxiety-related effects from Aβ and spatial-memory outcomes. EPRAP is an intracellular regulatory protein with cell-dependent functions, so its deletion is a different intervention from EP4 receptor blockade [6,55,56,57].

The EP4 evidence supports opposite contributions at the production and clearance sides of Aβ homeostasis: enhanced APP processing in some APP-expressing cells, and improved inflammatory control and Aβ uptake in microglia [6,40,53]. Representative experimental findings are summarised in Table 1.

## 6. Ageing and EP2-Associated Metabolic Changes in Myeloid Cells

Ageing alters PGE_2_–EP2 signalling and glucose utilisation in myeloid cells, linking receptor function to cellular metabolic state [5,13,42].

### 6.1. Age-Related Changes in PGE_2_–EP2 Signalling

The late-stage Aβ response to global EP2 deletion described in Section 4 establishes an age-dependent effect in APPswe/PS1ΔE9 mice [42]. Johansson et al. further found that Aβ42 oligomers induced a greater increase in EP2 expression and inflammatory responses in peritoneal macrophages from aged than from young mice. EP2 activation further enhanced this response while suppressing the expression of several chemokines and Aβ-degrading enzymes [5]. In ageing studies by Minhas et al., PGE_2_ levels were increased in the plasma and cortex of aged mice, together with higher EP2 expression in myeloid cells; related age-associated changes were also observed in monocyte-derived macrophages from older human donors [13]. Older myeloid cells thus encounter changes in both PGE_2_ availability and receptor responsiveness.

### 6.2. EP2-Mediated Glucose Diversion and Central and Peripheral Effects

Minhas et al. found that PGE_2_–EP2 signalling promotes the storage of glucose as glycogen in myeloid cells, reducing carbon flux into glycolysis and mitochondrial oxidation. Mechanistic experiments showed that EP2 activates Akt, promotes inhibitory phosphorylation of glycogen synthase kinase 3β (GSK3β), and thereby increases the activity of glycogen synthase 1 (GYS1). Reducing myeloid EP2 signalling decreased glycogen storage and restored glucose metabolic flux and mitochondrial respiration [13]. Liu et al. found that PF-04418948 increased respiratory capacity and adenosine triphosphate (ATP) production in human CD14^+^ monocytes and enhanced Aβ uptake, including in the classical subset. In 5×FAD mice, respiration assays used peripheral blood mononuclear cells (PBMCs), while monocyte analyses identified inflammatory, senescence-associated, and phagocytic abnormalities; treatment improved these measures [58]. The mouse PBMC respiration result is a mixed-cell measurement, whereas the uptake analyses resolve monocyte populations.

In aged mice, both the brain-penetrant antagonist compound 52 (C52) and predominantly peripheral PF-04418948 improved hippocampal plasticity and the memory measures assessed [13]. In 5×FAD mice, prolonged PF-04418948 treatment improved peripheral monocyte function alongside lower brain Aβ deposition and better behavioural performance [58]. The glycogen-partitioning mechanism was established in ageing models, whereas the 5×FAD findings primarily concern peripheral-cell function and AD-related outcomes. Together, these studies support peripheral myeloid function as a relevant component of systemic EP2 pharmacology.

The 5×FAD study also examined markers related to APP processing, Aβ degradation, and Aβ transport in the brain. No clear differences were found between treatment and control groups in the APP-processing enzymes, Aβ-degrading enzymes, or blood–brain barrier Aβ transport proteins examined, and colocalisation of Aβ with microglia did not indicate enhanced microglial uptake [58]. Monocyte depletion, lineage tracing, or Aβ flux measurements would help determine how much the peripheral response contributes to the reduced brain burden.

### 6.3. Metabolic and Cellular States of Microglia in AD

Ageing-related lipid storage offers a separate metabolic background for EP2 research. Marschallinger et al. identified lipid-droplet-accumulating microglia in aged mouse and human brains; the experimental cells showed impaired phagocytosis, increased reactive oxygen species, and inflammatory cytokine release [59].

Studies in AD models show that metabolic changes in microglia can directly influence Aβ handling and the surrounding tissue. Prakash et al. found that Aβ induced diacylglycerol O-acyltransferase 2 (DGAT2)-associated triglyceride synthesis and lipid-droplet accumulation, and that microglia with a higher lipid-droplet burden showed impaired Aβ phagocytosis. Pharmacological targeting of DGAT2 enhanced Aβ uptake and reduced plaque burden and neuronal injury in 5×FAD mice [60]. Studies using an antibody that activates triggering receptor expressed on myeloid cells 2 (TREM2) provide evidence from a different metabolic direction: enhanced TREM2 signalling improved mitochondrial metabolism in human iPSC-derived microglia, while in AD mouse models the intervention increased microglial responses and brain glucose metabolism [61]. These interventions link altered lipid storage or mitochondrial function to microglial responses through mechanisms distinct from EP2-dependent glycogen partitioning.

TREM2-dependent metabolism was established in a complementary loss-of-function study. Ulland et al. linked TREM2 deficiency to defective mechanistic target of rapamycin (mTOR) signalling, low ATP, and increased autophagy. Cyclocreatine restored plaque-associated microglial clustering and reduced adjacent neuronal dystrophy in TREM2-deficient amyloid-model mice [62].

Autophagy and mitochondrial quality control are also relevant to microglial metabolic regulation in AD. Autophagy–lysosomal abnormalities are among the broader disturbances in proteostasis and metabolism described in AD [63]. In BV2 cells, Guo et al. found that ciliatoside A enhanced AMPK/ULK1- and PINK1/Parkin-associated mitophagy and attenuated NLRP3 inflammasome activation. In 3×Tg-AD mice, systemic treatment reduced Aβ deposition and neuroinflammation and improved behavioural performance [64].

Mitochondrial damage and impaired clearance can also contribute to innate immune activation through mitochondrial DNA-associated pathways, including cGAS–STING signalling [65]. In 5×FAD mice, He et al. found that inducible microglia-specific cGAS deletion altered plaque-associated microglial responses and reduced Aβ pathology and neuritic injury, with improvements in the behavioural measures assessed [66]. These studies provide a basis for examining mitochondrial quality control and innate immune signalling alongside microglial metabolic changes in AD. Neither study directly investigated EP2 or EP4 signalling.

Human brain studies further show that AD microglia do not occupy a single stable metabolic or inflammatory state. Sun et al. analysed approximately 194,000 single-nucleus transcriptomic and epigenomic profiles of microglia from 443 individuals and identified multiple states associated with homeostasis, inflammation, and lipid handling, together with state-specific and AD stage-associated expression changes [67]. Haney et al. identified an acyl-CoA synthetase long-chain family member 1 (ACSL1)-associated lipid-related microglial state in human AD brain tissue that was enriched in individuals with APOE4/4. In human iPSC-derived microglia, fibrillar Aβ induced ACSL1 expression, triglyceride synthesis, and lipid-droplet accumulation in an APOE-dependent manner. Conditioned medium from microglia with a high lipid-droplet burden also induced tau phosphorylation and neurotoxicity [68].

Human AD tissue also contains glial populations with senescence-associated features. Fancy et al. combined imaging mass cytometry and single-nucleus sequencing and associated microglial senescence programs with amyloid burden, mitochondrial and stress-related changes, and reduced expression of phagocytic pathways [69].

Microglial expression patterns also vary with local pathology [67]. Using spatial transcriptomics and single-nucleus sequencing of brains from very old individuals, Lu et al. identified a spatial pathological transition from Aβ-associated inflammatory programs to tau-associated antigen-presentation programs and observed distinct pathology–cell response patterns in individuals with cognitive resilience [70]. The cross-sectional design maps regional and individual differences rather than following individual cells through a temporal transition.

A multiregion human atlas by Mathys et al. sampled six brain regions across 48 donors and identified cell-type- and region-specific responses to AD pathology. Such regional differences provide a reason to compare EP-receptor expression in anatomically defined cell populations [71].

The direct EP2 metabolic evidence concerns glucose partitioning, mitochondrial respiration, and peripheral myeloid-cell function [13,58]. AD studies describe additional changes in lipid storage, mitochondrial quality control, innate immune signalling, APOE-associated differences, and pathology-related microglial states [60,67,68,69,70]. Examining EP2 responses across defined microglial states, together with metabolic and Aβ-handling measurements, would help extend these findings to the cellular contexts of AD.

## 7. Pharmacological Development of Selective EP2 Antagonists

The development of selective EP2 antagonists has focused primarily on receptor selectivity, solubility, metabolic stability, and brain exposure [46,72,73]. Improvements in pharmacokinetic properties have enabled longer-term dosing, allowing studies to progress from acute inflammatory and neural injury models to AD models [47,48,58,74].

### 7.1. Optimisation of Receptor Selectivity and Pharmacokinetic Properties

TG6-10-1 was an early EP2 antagonist with measurable brain exposure, but its selectivity for EP2 over the D-prostanoid receptor 1 (DP1) was only approximately 10-fold, its aqueous solubility was approximately 27 μM, and its plasma half-life in rodents was approximately 1.7 h, limiting its use in chronic models. Subsequent structural optimisation yielded TG11-77. Conversion to the hydrochloride salt improved aqueous solubility while retaining brain penetrance, making the compound suitable for prolonged administration in drinking water [72]. In a separate chemical series, compound 52 showed single-digit nanomolar binding affinity and approximately 4000-fold selectivity over other EP receptors, together with favourable pharmacokinetic properties in rodents and good central nervous system (CNS) penetration. In ex vivo brain sections, compound 52 enhanced macrophage-mediated clearance of Aβ plaques [46].

Antagonist-bound receptor structures provide a molecular explanation for subtype selectivity. Wu et al. resolved EP2 with PF-04418948, EP4 with grapiprant, and both receptors with the dual antagonist TG6-129. Differences in ligand-pocket interactions provide a structural basis for further medicinal chemistry [75].

The original characterisation of PF-04418948 established competitive, surmountable EP2 antagonism, with a functional *K*_B_ of 1.8 nM in a human EP2 cAMP assay and oral antagonism of a butaprost-induced vascular response in rats. This receptor pharmacology should be distinguished from the peripheral distribution characterised in later ageing and AD studies [76].

TG8-260 further improved oral exposure, with a functional antagonist constant (*K*_B_) of approximately 13.2 nM, approximately 500-fold selectivity over the other prostanoid receptors tested, an oral bioavailability of 77.3%, and a plasma half-life of approximately 2.14 h after oral administration. However, absorption, distribution, metabolism, and excretion (ADME) profiling also identified substantial cytochrome P450 (CYP450) inhibition [73]. Development studies of TG11-77 similarly evaluated metabolic enzymes, drug transporters, and plasma protein binding. Inhibition of CYP2C8 and possible weak time-dependent inhibition of CYP3A4 were identified as potential drug–drug interaction liabilities requiring further evaluation [74].

More recent optimisation has addressed both antagonist potency and metabolic stability. CJ-18, CJ-19, and CJ-21 showed *K*_B_ values of approximately 5.0, 4.5, and 0.8 nM, respectively. All three were more stable than TG11-77 in human liver microsomes, whereas improved stability in mouse liver microsomes was most evident for CJ-18. These compounds reduced inflammatory mediator release from LPS-stimulated primary microglia and attenuated injury after oxygen–glucose deprivation in mixed neuron–glia cultures, although in vivo half-life and brain exposure have not yet been reported [77]. BPN-37440 has in vivo pharmacokinetic and brain-exposure data and efficacy after status epilepticus [78]. AD-model evidence is currently absent for both series [77,78].

### 7.2. Preclinical Efficacy and Translational Safety

The effects of EP2 antagonism on inflammation and tissue injury are not always concordant. In a rat model of status epilepticus, TG8-260 reduced hippocampal inflammation and glial responses but did not attenuate the neuronal injury or blood–brain barrier disruption that was measured [79]. In an LPS-induced encephalopathy model, TG6-10-1 reduced inflammation and synaptic protein loss and improved performance in novel object recognition and sucrose preference tests [80]. The drug effects on tissue and behaviour were therefore model-specific, even when inflammatory markers declined [79,80].

After status epilepticus, short-term TG11-77 treatment reduced delayed mortality and microglial responses and attenuated subsequent Y-maze abnormalities [74]. BPN-37440 similarly reduced microglial responses in the amygdala, cortex, and hippocampal CA3 region after short-term treatment and prevented memory deficits measured approximately 1–1.5 months after status epilepticus [78]. These are acute-injury treatment studies with later behavioural follow-up, rather than tests of sustained dosing during progressive AD pathology.

Initial safety studies in healthy rodents found no clear effects of TG6-10-1 or TG11-77.HCl on basal blood pressure, heart rate, or respiratory function. Chronic exposure to TG11-77.HCl likewise did not substantially alter blood cell counts, bone volume, or bone mineral density [81]. A 7-day dose-ranging study in rats found no clear clinical, organ-level, or histopathological toxicity [74]. TG11-77 remains a preclinical candidate; no publicly reported human trial was identified in this review.

At present, human data on selective EP2 antagonism are derived mainly from PF-04418948. The compound was evaluated in NCT01002963, a single-dose phase I safety and tolerability study in healthy volunteers, although no study results have been posted in the trial registry [82]. Varvel et al. later reported that PF-04418948 achieved the expected plasma exposure in humans but was accompanied by dose-related increases in bilirubin at exposures predicted to be pharmacologically active. These changes were attributed to inhibition of organic anion transporting polypeptides 1B1 and 1B3 (OATP1B1 and OATP1B3) and multidrug and toxin extrusion protein 1 (MATE1) [74]. These findings point to a transporter-related liability specific to PF-04418948 and do not demonstrate that EP2 blockade itself causes liver injury. Because AD treatment would likely require chronic administration in older adults, subsequent candidates will require repeated-dose safety studies and systematic assessment of drug–drug interaction risks.

### 7.3. Long-Term Intervention in AD Models

Long-term studies of TG11-77.HCl in AD have primarily used an LPS-plus-5×FAD two-hit model. In the 2021 study, treatment reduced cortical inflammatory transcription and glial markers in female mice, but plaque deposition was modestly increased. The same anti-inflammatory effect was not observed in 5×FAD mice without additional LPS exposure or in the LPS-only cohort [47]. A 2026 study used 5×FAD mice on a B6SJL genetic background together with a modified LPS dose and treatment schedule. Glial responses were reduced in both sexes, whereas reductions in inflammatory transcription and amyloid burden were observed mainly in males [48]. Genetic background, LPS exposure, and treatment schedule changed alongside the sex-specific results [47,48].

Behavioural effects in the 2026 study differed by task. Treated mice reached a within-group reduction in escape latency earlier during water-maze training, and probe-trial target-quadrant preference was most apparent in males. Training-phase learning did not differ significantly between treatment groups, and Y-maze alternation was unchanged [48]. The plaque, inflammatory, and behavioural endpoints therefore describe separate components of the treatment response [47,48].

Liu et al. treated male 5×FAD mice with PF-04418948 from 2 months of age for 4 months, without an additional LPS challenge. Treatment improved peripheral-cell respiration and monocyte Aβ uptake, reduced brain Aβ burden, and improved water-maze and novel-arm exploration performance [58]. Ageing studies had characterised PF-04418948 as predominantly peripheral [13]. The AD study found no increase in Aβ–microglia colocalisation. Peripheral monocytes were proposed to contribute to the benefit, although brain exposure was not excluded under blood–brain barrier disruption [58].

Central exposure and peripheral myeloid effects are therefore important variables when comparing the pharmacological effects of EP2 antagonists [13,58]. A direct comparison after pathology is established, with matched peripheral exposure and measured unbound brain concentrations, could help distinguish these contributions. Table 2 summarises the pharmacological properties, treatment regimens, CNS exposure, and available evidence for representative EP2 antagonists and EP4 modulators, distinguishing AD-related findings from ageing and other non-AD experimental evidence.

## 8. Key Issues in Therapeutic Translation

Figure 3 outlines the variables that need to be resolved when evaluating EP2- and EP4-directed interventions.

### 8.1. Cellular Sources of EP4 Effects and Aβ Kinetics

The available EP4 studies do not show whether changes in brain Aβ arise mainly from altered production or clearance [6,53]. In APP23 mice, global EP4 deletion or systemic antagonism reduced Aβ burden and altered APP processing [53], whereas myeloid EP4 deletion in APP-PS1 mice increased early inflammation and Aβ deposition [6]. Because these studies differed in disease model, animal age, and the cellular scope of intervention, direct comparisons are difficult. Comparisons within the same model, using age-matched cohorts and cell-type-specific interventions, would reduce these sources of confounding.

These contrasting findings complicate the therapeutic use of systemic EP4 agonists or antagonists. In microglial models, EP4 activation promotes Aβ uptake and limits inflammatory responses, whereas EP4 signalling in some APP-expressing cells facilitates APP internalisation and Aβ production [6,40,53]. Microglia-targeted delivery of EP4 agonists may reduce drug exposure in other APP-expressing cell populations. The ligand-dependent coupling properties of EP4 also provide a rationale for testing signalling-biased agonists [31]. Region-restricted administration may limit drug distribution but cannot provide cell-type selectivity. These approaches have yet to be tested for their ability to separate the different cellular effects of EP4 in AD models.

Adult-inducible neuronal and myeloid EP4 manipulation could be paired with measurements of APP processing and Aβ turnover. Uptake and degradation require separate assays: intracellular Aβ can increase through greater uptake or impaired degradation, whereas plaque burden reflects the accumulated consequences of Aβ production and clearance.

Human studies have established methods for assessing Aβ kinetics. Stable isotope labelling kinetics (SILK) has been used to distinguish changes in Aβ production and clearance after γ-secretase inhibition: treatment reduced the production rate without significantly changing the clearance rate [85]. A 2024 multicentre study of Posiphen in early AD also incorporated SILK into pharmacokinetic and pharmacodynamic assessment, although the prespecified analysis of the Aβ40 production rate did not show a significant treatment effect [86]. SILK therefore provides an established method for assessing Aβ production and clearance separately in EP-receptor intervention studies.

### 8.2. Distinguishing Central and Peripheral EP2 Actions

The cellular scope of genetic manipulation affects the interpretation of EP2 studies. In a status epilepticus model, Varvel et al. found that the CD11b-Cre strategy used in their study predominantly targeted circulating myeloid cells, with little recombination in microglia, while also affecting a subset of brain vascular endothelial cells [87]. Conditional EP2 deletion attenuated the IL-6 response and blood–brain barrier disruption but did not reduce the neuronal injury that was measured. In the same study, systemic EP2 antagonism also reduced monocyte entry into the brain [87]. These findings indicate that the actual cellular range of myeloid conditional deletion needs to be verified in each model and that the contribution of the neurovascular interface should also be considered.

Benefits of predominantly peripheral EP2 antagonism occur in ageing [13] and in 5×FAD mice [58]. These observations motivate a direct test of peripheral myeloid contributions to brain pathology.

Brain-penetrant and predominantly peripheral antagonists should be compared at matched unbound plasma exposure and functional EP2 inhibition. Disease-related blood–brain barrier changes can alter distribution; this possibility was retained in the PF-04418948 AD study [58]. Measuring unbound brain exposure alongside lineage-specific receptor manipulation would identify the central contribution more directly.

### 8.3. Effects of Age, Pathological Stage, and Sex

The metabolic response to EP2 inhibition provides a reason to stratify experiments by age [13]. The EP4 deletion study additionally illustrates the difficulty of separating stage and sex when early and late cohorts use different sexes [6]. Studies should define pathological stage by baseline pathology as well as chronological age and treatment timing.

TG11-77 inflammatory effects were most apparent in females in 2021 [47], while the later study found male-predominant transcriptional and plaque responses [48]. A sex effect requires a treatment-by-sex interaction test; significance in one sex alone is insufficient. Prespecified analyses of drug intake and unbound exposure would help separate pharmacokinetic differences from receptor biology.

### 8.4. Functional Validation of EP Receptors in Human-Derived Systems

The regional *PTGER4* expression differences identified in human microglial profiling [54] and the state diversity mapped in AD [67] provide a sampling framework for functional studies. Functional validation requires paired receptor-protein and agonist-response measurements to link expression differences to EP signalling.

In iPSC-derived microglia–neuron co-cultures, manipulating *PTGER2* or *PTGER4* separately in each cell type would distinguish neuronal production from microglial uptake and degradation. Parallel neuronal-injury assays would then test whether altered Aβ handling protects neighbouring cells.

Human-derived models can also distinguish metabolic readouts from phagocytic function. In TREM2 R47H iPSC-derived microglia, Vasilopoulou et al. found that succinate was more effective at restoring oxidative phosphorylation, whereas citrate more effectively corrected Aβ phagocytic deficits. Separate functional assays therefore remain necessary when testing EP2-related metabolic rescue [88].

Tau evidence is much narrower than the Aβ evidence. In APP/PS1 mice and N2a cells, increased Ca^2+^ and mPGES-1/PGE_2_ were linked to EP1-, EP2-, and EP3-associated cyclin-dependent kinase 5 (CDK5) activation and tau phosphorylation; EP4 blockade did not produce the same response [89]. Independent tau models and cell-specific human-derived assays would test whether this reflects direct neuronal signalling or a microglial paracrine mechanism.

### 8.5. Translational Evaluation from Drug Exposure to Disease Outcome

Translational studies need exposure measurements linked to functional receptor inhibition. Unbound plasma and brain concentrations can be measured in animals; human CSF concentrations provide indirect evidence of CNS exposure. Receptor-stimulated responses in peripheral monocytes are candidate pharmacodynamic assays. Because both EP2 and EP4 generate cAMP [30], the assay requires receptor-selective controls and validation against drug concentration and sampling time.

Peripheral-cell respiration and monocyte Aβ uptake are candidate downstream functional endpoints [13,58]. PGE_2_ and its metabolites describe ligand availability and turnover. Positron emission tomography (PET) measurements using translocator protein (TSPO), purinergic P2X7 receptor (P2X7R), or colony-stimulating factor 1 receptor (CSF1R) report different inflammation-associated molecular processes [90]. These measures would complement a receptor-function assay rather than quantify EP2 activity directly.

SILK could connect receptor inhibition to Aβ turnover [85,86]. Axonal, synaptic, and cognitive measures are needed alongside Aβ kinetics to assess tissue and functional benefit. The dissociation among inflammatory, plaque, and behavioural results in TG11-77 studies [47,48] makes separate assessment of these endpoints essential for dose selection and intervention timing.

## 9. Conclusions

EP2 and EP4 link PGE_2_ signalling to different components of AD pathology. Their shared G_s_–cAMP pathway is modified by G-protein coupling, receptor trafficking, and microglial activation state. Changes in PGE_2_ synthesis, transport, and tissue or CSF concentrations further alter the ligand environment in which these receptors operate.

The most consistent evidence for EP2 inhibition concerns improved myeloid Aβ handling and reduced paracrine neuronal injury. Ageing studies add a mechanism involving glucose partitioning and respiration, with a substantial peripheral component. EP4 has contrasting cellular actions: microglial signalling supports inflammatory control and Aβ uptake, whereas some APP-expressing cells show increased APP processing and Aβ production. Chronic EP2 antagonist studies have progressed beyond acute inflammation models, but their plaque and behavioural outcomes vary with model, treatment schedule, and sex.

The first priority is to establish cell-specific EP2 and EP4 effects in AD models, combining receptor manipulation with measured drug exposure and separate assessments of Aβ production and clearance. For EP2, the principal question is how central and peripheral inhibition contribute to tissue protection after pathology is established. For EP4, it is whether production- and clearance-related actions can be separated sufficiently to define a useful intervention.

## Figures and Tables

**Figure 1:**
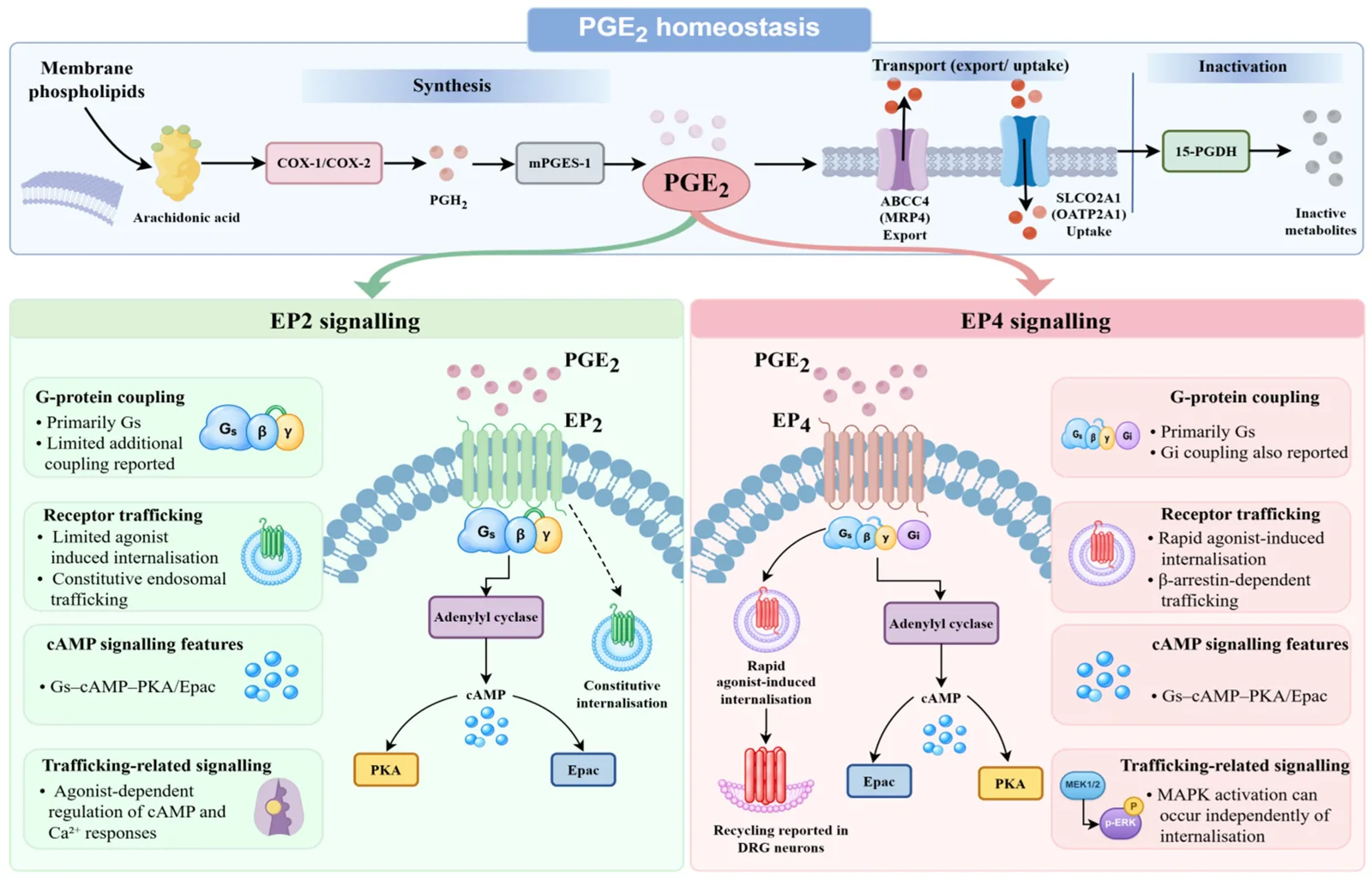
EP2 and EP4 both activate G_s_–cAMP signalling but differ in G-protein coupling and receptor trafficking. Blue, green, and pink shading denote PGE_2_ homeostasis, EP2 signalling, and EP4 signalling, respectively. Solid arrows indicate the direction of the depicted processes, whereas the dashed arrow in the EP2 panel denotes constitutive receptor internalisation. EP2 exhibits limited agonist-induced internalisation but undergoes constitutive internalisation through a dynamin-dependent, β-arrestin-independent pathway, with trafficking to early and very early endosomes. EP4 undergoes rapid agonist-induced internalisation through mechanisms involving β-arrestin, dynamin, and clathrin-coated pits. Constitutive EP2 trafficking differentially modulates agonist-evoked cAMP and Ca^2+^ responses, whereas EP4-mediated MAPK activation is preserved when receptor internalisation is inhibited. Recycling of internalised EP4 to the plasma membrane has been reported in cultured dorsal root ganglion neurons. These trafficking and signalling observations derive mainly from non-microglial experimental systems. By Figdraw. Zhao, X. (2026). https://www.figdraw.com/. Authorization code: PRYWIbd17d.

**Figure 2:**
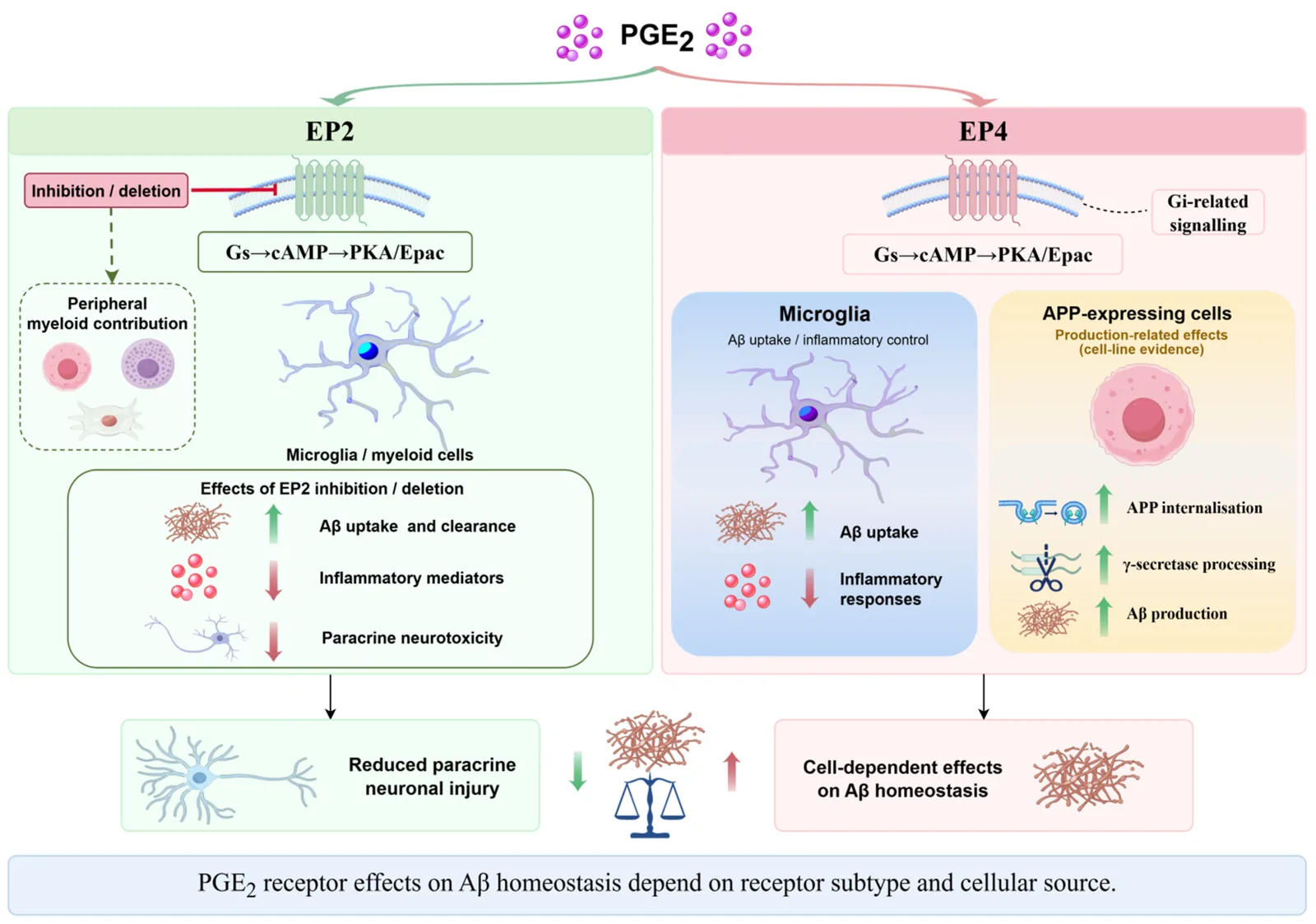
Cell-dependent effects of EP2 and EP4 on Aβ homeostasis. Green and pink backgrounds distinguish the EP2 and EP4 branches, respectively; blue and yellow backgrounds within the EP4 branch distinguish microglia and APP-expressing cells. The blunt-ended red line denotes EP2 inhibition/deletion. The dashed connectors link EP2 inhibition/deletion to the peripheral myeloid contribution and EP4 to Gi-related signalling. EP2 inhibition or deletion enhances myeloid Aβ uptake and clearance and reduces paracrine neuronal injury in experimental systems; peripheral myeloid cells may also contribute to the response to systemic antagonism. EP4 activation supports Aβ uptake and inflammatory control in microglia, whereas EP4 signalling in some APP-expressing cells promotes APP internalisation and Aβ production. Upward and downward arrows indicate the direction of the effects depicted, not their magnitude. Abbreviations: Aβ, amyloid-β; APP, amyloid precursor protein; cAMP, cyclic adenosine monophosphate; Epac, exchange protein directly activated by cAMP; PKA, protein kinase A. By Figdraw. Zhao, X. (2026). https://www.figdraw.com/. Authorization code: STWOA87474.

**Figure 3:**
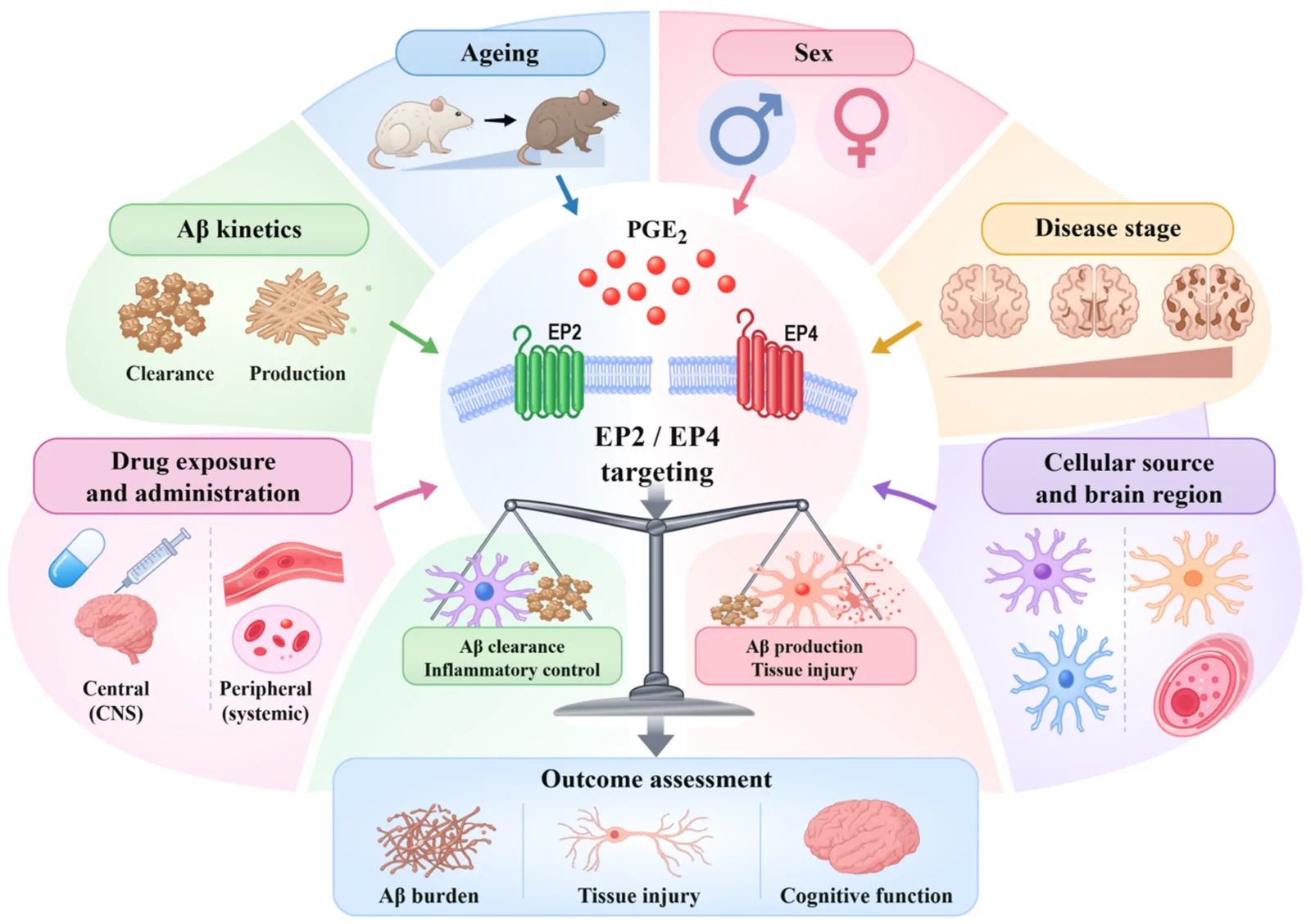
Variables and outcomes in the evaluation of EP2- and EP4-directed interventions. Age, sex, disease stage, cellular source and brain region, drug exposure, and the balance between Aβ production and clearance affect the interpretation of receptor-targeting studies. Central and peripheral drug actions should be assessed alongside Aβ burden, tissue injury, and cognitive outcomes. The diagram is a conceptual framework rather than a quantitative model. Abbreviations: Aβ, amyloid-β; CNS, central nervous system; PGE_2_, prostaglandin E_2_. By Figdraw. Zhao, X. (2026). https://www.figdraw.com/. Authorization code: ASWWUd0d04.

**Table 1:** Representative experimental evidence for EP2 and EP4 in AD-related processes.

Process	Intervention and Model	Main Finding	Evidence Level and Interpretation	Reference
APP expression	Pharmacological receptor manipulation; primary rat microglia	PGE_2_ and EP2 activation increase full-length APP through a cAMP–PKA-related mechanism	APP expression study; Aβ production was not measured directly	[41]
Aβ production	Pharmacological receptor manipulation; mutant APP-expressing cells	PGE_2_ increases γ-secretase activity and Aβ release; EP2/EP4 contribution varies with cellular context	Cellular APP-processing mechanism	[40]
Brain Aβ accumulation	Global EP2 deletion; APP transgenic mice	Brain Aβ and plaque burden are reduced	Global genetic manipulation; production and clearance contributions were not separated	[42]
Aβ phagocytosis and ex vivo clearance	EP2 deletion; primary mouse microglia and human AD brain sections	Increased Aβ phagocytosis and clearance of deposits from brain sections	Direct microglial functional evidence	[12]
CD36-associated phagocytosis	Genetic or pharmacological EP2 inhibition; primary microglia	Increased CD36 expression and partial restoration of Aβ42 phagocytosis after TLR stimulation	Links an Aβ-recognition receptor to uptake function	[43]
Recruitment to lesions	Global EP2 deletion; APP-PS1 mice	Increased microglial recruitment around early plaques	Lesion-localisation evidence; does not by itself measure clearance rate	[5]
Clearance of injected Aβ	Myeloid EP2 conditional deletion; labelled Aβ injected into aged mouse cortex	Reduced residual Aβ after injection	Acute in vivo clearance assay; distinct from chronic plaque outcome	[5]
Synaptic and memory outcomes	Myeloid EP2 conditional deletion; APP-PS1 mice	Reduced synaptic injury and memory deficits	Functional disease-model outcome; intervention involves myeloid cells	[5]
Paracrine neurotoxicity	Microglial EP2 deletion; microglia–neuron co-culture	Reduced neurotoxicity after Aβ or LPS stimulation, involving COX-2, iNOS, and reactive oxygen species	Direct neuronal-injury endpoint	[12,49]
Microglial death	EP2 activation or antagonism; cultured rat microglia	Modulation of ROS- and caspase-associated delayed cell death	Regulation of activated microglial survival; relevance to AD remains uncertain	[51]
Inflammatory regulation	EP4 activation and myeloid conditional deletion; LPS model	EP4 activation reduces inflammation, whereas conditional deletion increases inflammation and lipid peroxidation	Non-AD inflammatory model; includes central and peripheral myeloid effects	[52]
Aβ uptake	EP4 activation; BV2 cells	Increased uptake of labelled Aβ42	Cellular uptake assay; long-term in vivo clearance not measured	[6]
Inflammation and Aβ deposition	Myeloid EP4 conditional deletion; APP-PS1 mice	Increased early inflammation, oxidative injury, and Aβ deposition	Early- and late-stage cohorts also differ in age and sex	[6]
APP processing and tissue outcomes	Global EP4 deletion or antagonism; APP23 mice	Reduced Aβ, plaque burden, neuronal and synaptic injury; improved performance on the cognitive measures assessed	Global intervention; cellular source of benefit not established	[53]
Aβ degradation and transport-related markers	PF-04418948 treatment; 5×FAD mice	No clear changes in the Aβ-degrading enzymes or blood–brain barrier Aβ transport proteins examined	Marker measurements do not directly quantify Aβ degradation or transport flux	[58]
EPRAP-associated inflammation	Global EPRAP deletion; J20 mice	Reduced microglial accumulation and inflammatory transcription; no significant change in Aβ or spatial memory; abnormal anxiety-like behaviour corrected	EPRAP manipulation cannot substitute for EP4 receptor manipulation	[57]
Ageing-related metabolism	Myeloid EP2 deletion or antagonism; aged mice and myeloid cells	Reduced glycogen storage, restored glucose flux and mitochondrial respiration, with improvement in ageing-related functional outcomes	Ageing model; not yet established as an AD-specific mechanism	[13]

Abbreviations: AD, Alzheimer’s disease; Aβ, amyloid-β; APP, amyloid precursor protein; CD36, cluster of differentiation 36; COX-2, cyclooxygenase-2; EPRAP, EP4 receptor-associated protein; EP, E-prostanoid receptor; iNOS, inducible nitric oxide synthase; LPS, lipopolysaccharide; ROS, reactive oxygen species; TLR, Toll-like receptor.

**Table 2:** Representative EP2 and EP4 pharmacological modulators relevant to neuroinflammation and Alzheimer’s disease.

Compound and Action	Potency and Selectivity	Model and Regimen	CNS Exposure and Experimental Findings	Key Limitations
EP2 antagonists				
TG6-10-1; antagonist	*K*_B_ = 17.8 nM; ≥300-fold selective over EP3, EP4, and IP, but 10-fold over DP1 [83].	Non-AD SE: 5 mg/kg i.p. at 4, 21, and 30 h; LPS encephalopathy [80,83].	Brain/plasma ratio 1.6; reduced inflammation in non-AD CNS models [80,83].	Limited DP1 selectivity, low solubility, and short half-life.
Compound 52 (C52); antagonist	Single-digit-nanomolar affinity; approximately 4000-fold selective over other EP receptors [46].	AD tissue ex vivo; aged mice: 10 mg/kg/day orally for 1 month [13,46].	CNS-permeable; increased plaque clearance ex vivo and improved myeloid metabolism and memory in aged mice [13,46].	No chronic in vivo AD study; ageing evidence is not AD-specific.
TG8-260; antagonist	*K*_B_ = 13.2 nM; >500-fold selective over tested prostanoid receptors [73].	Non-AD rat SE: 25 mg/kg i.p. at 2, 8, and 20 h [79].	Oral bioavailability 77.3%; brain/plasma ratio 0.02–0.05; no direct AD evidence [73,79].	Limited CNS distribution; CYP inhibition.
TG11-77.HCl; antagonist	*K*_B_ = 9.7 nM; >300-fold selective over other prostanoid receptors [72].	LPS-plus-5×FAD mice; drinking water for 8–12 weeks; nominal 100 mg/kg/day [47,48].	Brain/plasma ratio 0.4 to >1 [72,74]; reduced glial activation, but amyloid and behavioural effects varied by model and sex [47,48].	No human trial; potential CYP-mediated interactions.
PF-04418948; antagonist	*K*_B_ = 1.8 nM; IC_50_ = 16 nM; >2000-fold functionally selective [76].	Aged mice: 2.5 mg/kg/day orally for 6 weeks [13]. Male 5×FAD mice: 3 mg/kg/day i.p. for 4 months [58]. Phase I single-dose study, NCT01002963 [82].	Predominantly peripheral in aged mice; reduced Aβ burden and improved behaviour in 5×FAD mice [13,58].	Brain exposure not measured in 5×FAD; trial results not posted; bilirubin/transporter liability [74,82].
CJ-18, CJ-19, and CJ-21; antagonists	*K*_B_ = 5.0, 4.5, and 0.8 nM; ≥700-fold selective over EP4, DP1, and IP [77].	LPS-treated primary microglia (10 μM); mixed neuron–glia OGD cultures [77].	Brain exposure not reported; no direct AD evidence.	Cellular evidence only; no in vivo PK or chronic safety data.
BPN-37440; antagonist	Human EP2 *K*_B_ = 5.65 nM; mouse EP2 *K*_B_ = 9.55 nM; >175-fold selective [78].	Non-AD mouse SE: 10 mg/kg i.p. (short course) [78].	Brain/plasma *K*_p_ = 0.29–0.76; prevented delayed memory deficits after SE [78].	No AD-model or chronic-treatment evidence.
EP4 modulators				
ONO-AE1-329 (AE1-329); agonist	*K*_i_ = 9.7 nM at EP4; >1 μM at other EP receptors [6].	Aβ_42_-stimulated primary microglia/BV2 cells; 100 nM (6-h expression assay; 3-h uptake pretreatment) [6].	Reduced inflammatory responses and increased cellular Aβ uptake; systemic brain exposure not reported [6].	Cellular uptake does not establish long-term clearance; no chronic AD study.
ONO-AE3-208 (AE3-208); antagonist	*K*_i_ = 1.3 nM at EP4 and 30 nM at EP3 [53].	APP23 mice; supplemented chow from 3 to 6 months, mean intake 17.8 mg/kg/day [53].	Reduced brain Aβ burden, APP internalisation, and γ-secretase activation; brain exposure not measured [53].	Responsible cell type unresolved; ~23-fold selectivity over EP3 warrants caution.
L-902,688; agonist	*K*_i_ = 0.38 nM; EC_50_ = 0.6 nM; >4000-fold selective [84].	Non-AD rat transient MCAO: 0.3 or 1.0 mg/kg i.v. at reperfusion [84].	Brain exposure not reported; reduced BBB damage and inflammatory responses in a non-AD model [84].	Acute stroke and BBB disruption limit relevance to chronic AD.

Abbreviations: AD, Alzheimer’s disease; Aβ, amyloid-β; APP, amyloid precursor protein; BBB, blood–brain barrier; CNS, central nervous system; CYP, cytochrome P450; DP1, D-prostanoid receptor 1; EC_50_, half-maximal effective concentration; EP, E-prostanoid receptor; HCl, hydrochloride; IC_50_, half-maximal inhibitory concentration; i.p., intraperitoneal; i.v., intravenous; IP, prostacyclin receptor; *K*_B_, functional antagonist equilibrium constant; *K*_i_, inhibition constant; *K*_p_, brain-to-plasma partition coefficient; LPS, lipopolysaccharide; MCAO, middle cerebral artery occlusion; OGD, oxygen–glucose deprivation; PK, pharmacokinetics; SE, status epilepticus. Reported brain/plasma ratios do not establish unbound brain exposure or receptor occupancy. The ageing and non-AD findings are not evidence of efficacy in AD models.

## Data Availability

No new data were created or analyzed in this study. Data sharing is not applicable to this article.

## Abbreviations

AD	Alzheimer’s disease
ADME	Absorption, distribution, metabolism, and excretion
Aβ	Amyloid-β
APP	Amyloid precursor protein
ATP	Adenosine triphosphate
cAMP	Cyclic adenosine monophosphate
CNS	Central nervous system
COX	Cyclooxygenase
CSF	Cerebrospinal fluid
DGAT2	Diacylglycerol O-acyltransferase 2
EP	E-prostanoid receptor
Epac	Exchange proteins directly activated by cAMP
EPRAP	EP4 receptor-associated protein
GSK3β	Glycogen synthase kinase 3β
iNOS	Inducible nitric oxide synthase
iPSC	Induced pluripotent stem cell
LPS	Lipopolysaccharide
MAPK	Mitogen-activated protein kinase
MCI	Mild cognitive impairment
mPGES-1	Microsomal prostaglandin E synthase-1
NF-κB	Nuclear factor κB
PBMC	Peripheral blood mononuclear cell
PET	Positron emission tomography
PGE_2_	Prostaglandin E2
PGT	Prostaglandin transporter
PK	Pharmacokinetics
PKA	Protein kinase A
ROS	Reactive oxygen species
SILK	Stable isotope labelling kinetics
TREM2	Triggering receptor expressed on myeloid cells 2
